# Gemcitabine-cisplatin chemotherapy plus anti-PD-L1 therapy reinvigorates antitumor immune response by reprogramming the intrahepatic cholangiocarcinoma microenvironment

**DOI:** 10.3389/fimmu.2025.1666393

**Published:** 2025-12-02

**Authors:** Yang Song, Shao-wei Huang, Bo Shu, Ying-Xia Zhou, Wei-Dong Dai, Bao-Ye Sun

**Affiliations:** 1 Clinical Immunology Research Center, The Second Xiangya Hospital of Central South University, Changsha, Hunan, China; 2 Department of General Surgery, The Second Xiangya Hospital of Central South University, Changsha, Hunan, China; 3 Department of Surgical Operation, The Second Xiangya Hospital of Central South University, Changsha, Hunan, China

**Keywords:** intrahepatic cholangiocarcinoma, single-cell RNA-seq, tumor ecosystem, chemotherapy, immunotherapy

## Abstract

**Purpose:**

Gemcitabine-cisplatin chemotherapy combined with anti-PD-L1 (GCP) therapy exhibits potent antitumor efficacy in patients with advanced intrahepatic cholangiocarcinoma (ICC). We aim to determine the intra-tumoral changes of ICC following GCP therapy in this study.

**Methods:**

We performed single-cell RNA-seq (scRNA-seq) of 15 samples from 3 ICC patients receiving GCP therapy. The major findings of scRNA-seq analyses were further validated via analyzing the bulk RNA-seq data from the FU-iCCA cohort (n=244), as well as performing immunohistochemistry (IHC) and multiplex immunofluorescence (mIF) staining on a treatment-naïve tissue microarray (TMA) cohort (n=89) and a GCP-treated cohort (n=32).

**Results:**

For the scRNA-seq cohort, two patients achieved tumor regression and underwent liver resection after GCP combination therapy. The intra-tumoral enrichment of CCL18^+^ macrophages correlated with poor prognosis of ICC patients after curative resection in the TMA cohort. Reduced fractions of CCL18^+^ and SPP1^+^ macrophages were observed in the GCP-treated ICC specimens which achieved pathological response. Our scRNA-seq analyses revealed significant alterations in the tumor microenvironment following GCP therapy: tumor-infiltrating macrophages underwent a distinct antitumor phenotypic shift, transitioning from M2 toward M1 polarization; concurrently, CD8^+^ T cells exhibited enhanced costimulatory signaling characterized by CD81 upregulation and malignant cells demonstrated diminished immune escape characteristics alongside heightened activity in immune response-related pathways.

**Conclusions:**

Our preliminary findings reveal a generally reactivated antitumor immune response in ICC following GCP therapy, which could partly illuminate the enigmatic black box of intra-tumoral cellular states associated with treatment response.

## 1 Introduction

Intrahepatic cholangiocarcinoma (ICC) is a highly lethal malignancy accounting for up to 20% of all liver cancers (1), with continuously rising incidence and low 5-year survival rate (~9%) (2, 3). Surgical resection remains the only potential cure for ICC, whereas most patients are diagnosed at the advanced stage and only 20%-30% of patients are eligible for surgery (4). For patients with advanced ICC, palliative gemcitabine and cisplatin (GC) chemotherapy has been recommended as the first-line regimen since year 2010, but the median survival remains limited to approximately 1 year (5). Thus, identifying effective treatment strategies for advanced ICC is urgent, with high potential to increase the long-term survival of ICC patients. While clinical evidence suggested that immune checkpoint blockades (ICBs) like anti-PD-L1/PD-1 therapy alone could barely induce a higher objective response in patients with advanced ICC compared with standard GC chemotherapy (6, 7), the combination of GC and anti-PD-L1/PD-1 therapy (GCP) achieved robust and sustained overall survival benefit (8, 9), opening a new era of first-line treatment for advanced cholangiocarcinoma. However, how GCP therapy reshapes the ICC microenvironment and the specific response or resistance mechanisms demand further exploration.

Using single-cell RNA sequencing (scRNA-seq) and spatial transcriptomics (ST), numerous studies have depicted the single-cell landscape of liver cancer, including primary treatment-naïve HCC, early-relapse HCC, and primary ICC (10–15), as well as tumor cell evolution in response to ICBs (16, 17). Nevertheless, the majority of these studies have focused on treatment-naïve samples, and there have been few reports on single-cell sequencing of tumor samples after systemic therapy (18, 19). Particularly, the single-cell atlas of ICC after GCP therapy has not been well characterized. The underlying cellular phenotypic changes in the ICC ecosystem following combined GCP therapy remain largely elusive. Therefore, in-depth profiling of ICC specimens pretreated with GCP at the single-cell resolution could enhance our understanding of the cellular landscape related to treatment response and may aid in discovering more effective therapeutic strategies.

In this study, we performed scRNA-seq analyses of ICC samples collected before and after GCP treatment and depicted the intra-tumoral changes of diverse cell types in response to GCP therapy. Notably, we found that GCP therapy led to the phenotype transformation of tumor-infiltrating macrophages from M2 toward M1, the transformation of CD8^+^ T lymphocytes into co-stimulatory functional phenotypes, and the transformation of tumor cells from the classical malignant phenotypes to the inflammatory activation state. These findings in part uncover the reactivated antitumor immune landscape of ICC reprogrammed by the GCP therapy at a single-cell level.

## 2 Materials and methods

### 2.1 Patient samples

A total of 15 samples of tumor, adjacent para-tumor tissues, and peripheral blood from three ICC patients receiving GCP therapy were collected for scRNA-seq, including three pretreated tumor biopsies and two GCP-treated, surgically resected tumor samples. This study was approved by the Research Ethics Committee of the Second Xiangya Hospital of Central South University and complied with all relevant ethical standards. Written informed consent was obtained from all patients before systemic therapy or surgery.

### 2.2 Systemic therapy and treatment response evaluation

For advanced ICC patients, envafolimab (anti-PD-L1 antibody) combined with gemcitabine and cisplatin was administered on a 21-day cycle for four to six cycles. Envafolimab (150 mg) was administered subcutaneously once every week, in combination with gemcitabine (1,000 mg/m^2^) and cisplatin (25 mg/m^2^), which were administered intravenously on days 1 and 8 of each cycle. Patients were monitored by routine blood tests prior to treatment every 3 weeks and abdominal magnetic resonance imaging (MRI) and chest computed tomography (CT) every 2 months. Tumor responses were evaluated according to the modified Response Evaluation Criteria in Solid Tumors (mRECIST) (20). Partial response (PR) was defined as the sum of tumor diameters were reduced by ≥30% from baseline.

### 2.3 Preparation of single-cell suspension

Peripheral blood samples were collected prior to treatment or surgery in EDTA anticoagulant tubes. Fresh tumor samples were placed into RPMI 1640 medium (Gibco) with 10% fetal bovine serum (Gibco). The tissues were subsequently dissociated into single cells using the gentleMACS Dissociator (Miltenyi Biotec) following the steps detailed previously (10). After filtering through cell strainers and lysing red blood cells, the original unsorted single-cell suspensions were used for single-cell RNA sequencing.

### 2.4 Single-cell RNA sequencing

Following single-cell isolation, scRNA-seq was performed using a 10× chromium single-cell platform according to the manufacturer’s protocol. Briefly, scRNA-seq libraries were generated using the Chromium Single Cell 5' Library & Gel Bead Kit (10x GENOMICS) and sequenced on an Illumina NovaSeq 6000 sequencer.

### 2.5 ScRNA-seq data processing

Cell Ranger (v3.1.0) was applied for processing raw fastq files, read mapping, and gene expression quantification. The DoubletFinder algorithm was performed to remove doublets (21), and cells with less than 200 or more than 4,000 genes or >10% mitochondria genes were excluded. Harmony was used to integrate all samples and diminish batch effects (22). We used Seurat (v4) (23) to identify highly variable genes (n=2400) and perform principal component analysis (PCA) and cell clustering analysis visualized by the UMAP method. For the clustering of all cells, the top 20 PCs were selected with a resolution parameter equal to 0.8. The top 12 PCs with a resolution of 0.6 were used for T cells. For the clustering of myeloid cells, the top 16 PCs with a resolution parameter of 0.6 were selected. The labeling of cell types was performed manually according to the highly expressed marker or functional genes. Inferred copy number variation (CNV) analysis (24) was conducted to distinguish malignant cells with endothelial cells set as the normal reference. We used R packages Seurat (V4.1.0), ggplot2 (V3.3.5), ggpubr (V0.4.0), ggsignif (V0.6.3), patchwork (V1.1.1), EnhancedVolcano (V1.12.0), and pheatmap (V1.0.12) to visualize our data.

### 2.6 Intercellular ligand–receptor analysis

We utilized the CellChat R package (25) to infer the ligand–receptor interactions between different cell types. Differential cell–cell interaction analysis and specific signaling pathways were also visualized by CellChat.

### 2.7 Differential gene expression and pathway enrichment analysis

Differential gene expression analysis of cell populations was performed using the “FindMarkers” function (log2-scaled fold change ≥0.585 and P value < 0.01). The differentially expressed genes (DEGs) were visualized using the EnhancedVolcano package. We then performed pathway enrichment analysis for those DEGs using clusterProfiler V 4.2.2 (26) (enrichGO and enrichKEGG function) and gene set enrichment analysis (GSEA) (27) using the fgsea R package to analyze the different Hallmark gene sets in malignant cells.

### 2.8 Definition and calculation of gene signature scores

Gene signature scores were calculated based on the scRNA-seq data. For macrophages, phenotypic signatures such as M1 polarization and M2 polarization were described before (28). Other functional gene signatures for phagocytosis (GO:0006909), angiogenesis (GO:0001525), and complement (Hallmark) were derived from MSigDB (https://www.gsea-msigdb.org). For CD8^+^ T cells, T-cell phenotypes were determined based on gene signatures from a previous study (10). Specifically, tissue resident markers (RUNX3, NR4A1, NR4A3, CXCR6, CD69), exhausted markers (CTLA4, PDCD1, TIGIT, LAG3, HAVCR2), and co-stimulatory molecules (ICOS, CD28, TNFRSF14, TNFRSF25, TNFRSF9, and CD226) were used to define the tissue resident, exhausted, and co-stimulatory signatures, respectively. For malignant cells, gene sets related to immune surveillance (HLA-A, HLA-B, HLA-C, MICA, MICB) and immune escape (CD274, CD47, ADAM10, HLA-G, FASLG, CCL5, TGFB1, IL10, PTGER4) were used. For each gene signature, the “AddModuleScore” function in Seurat was used to compute the average scores of selected genes for individual cells.

### 2.9 Tissue microarray, immunohistochemistry, and immunofluorescence

For the tissue microarray (TMA) cohort, formalin-fixed and paraffin-embedded (FFPE) tumor specimens from 89 treatment-naïve patients who underwent curative resection for ICC were selected. All cases were pathologically diagnosed as ICC. For the GCP cohort, we collected surgically resected ICC samples from 32 patients who received curative resection after GCP therapy. For pathological assessments, patients achieving complete or major pathologic responses (≤10% viable tumor cells) were defined as pathologic responders. Immunohistochemistry (IHC) staining was performed as described before (29). In brief, tissue sections were baked, dewaxed, rehydrated, and blocked endogenous peroxidase activity by 3% H_2_O_2_. Antigen retrieval was conducted using sodium citrate buffer (pH=6) in microwave, and non-specific binding sites were blocked by 10% goat serum. Next, the slides were incubated with primary antibodies including CRP (1:2,000 dilution, ab32412, Abcam) at 4°C overnight. After washing, the slides were incubated with goat anti-mouse or anti-rabbit secondary antibody and visualized with DAB solution. We performed immunofluorescence (IF) staining following the same steps for IHC before incubation of antibodies. For CCL18^+^ CD68^+^ macrophages, the sections were incubated with CD68 primary antibody (1:400 dilution, Cat# 76437, CST), followed by an anti-rabbit Alexa Fluor 647-conjugated secondary antibody (1:400 dilution, B40958, Thermo Fisher). Then, the slides were incubated with a CCL18 primary antibody (1:100 dilution, ab104867, Abcam), and a second incubation in Alexa Fluor 488 donkey anti-rabbit antibody (1:400 dilution, A21206, Thermo Fisher). For CD81^+^ CD8^+^ T cells, the slides were incubated with CD8 primary antibody (1:2,000 dilution, ab237709, Abcam), followed by Alexa Fluor 647-conjugated secondary antibody as mentioned above. After that, the slides were incubated with CD81 primary antibody (1:200 dilution, A01281-2, BOSTER) and Alexa Fluor 488 donkey anti-rabbit antibody as described above. The staining of SPP1^+^ CD68^+^ macrophages was carried out as reported previously (17).

All immune stained slides were scanned and evaluated by two investigators blinded to patient characteristics using the CaseViewer 2.3 (3DHISTECH). The score for IHC staining intensity was quantified as 0 for negative, 1 for weak, 2 for moderate, and 3 for strong signal. A positive IHC staining was defined as a weak to strong staining pattern (score 1 to 3). As for the dual IF staining of CCL18^+^ CD68^+^ macrophages and CD81^+^ CD8^+^ T cells, five fields (400×) of immune cell-enriched tumor area from each dot in tissue microarray were selected and double-stained cells were counted manually.

### 2.10 Statistical analysis

Statistical analyses were performed using R version 4.1.2 and GraphPad Prism (v.9.0). Comparisons of gene signature scores and cellular proportions of selected cell types between two groups were conducted using unpaired two-tailed Wilcoxon rank-sum test. Comparisons of cell-type proportions of paired tumor and adjacent non-tumor tissues were performed using paired two-tailed Wilcoxon rank-sum tests. Kaplan–Meier survival analyses were performed, and log-rank test p values were calculated by R packages survival and survminer. P < 0.05 was considered as statistically significant.

## 3 Results

### 3.1 A single-cell atlas of the tumor niche in primary pretreated and GCP-treated ICC

**Figure 1:**
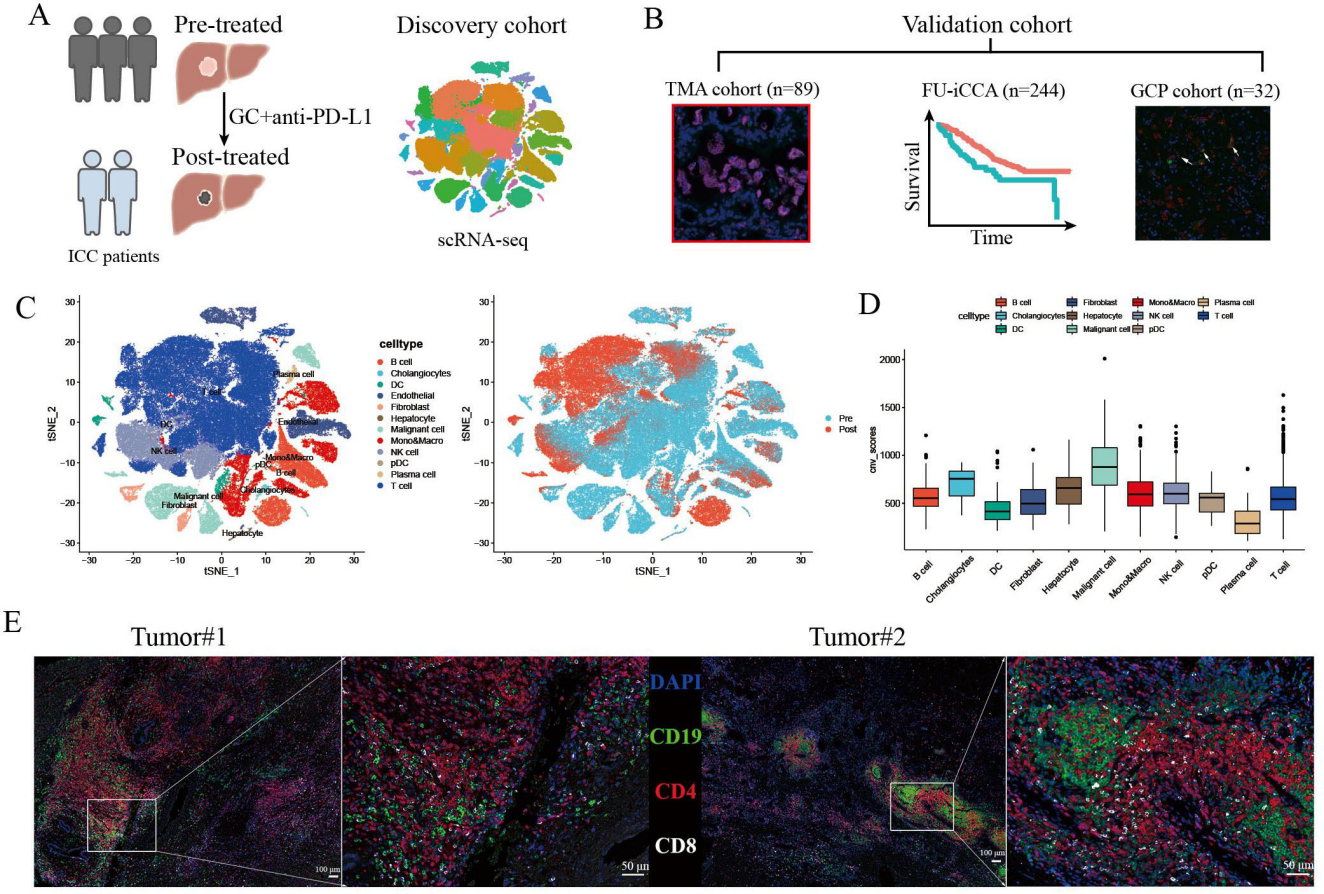
ScRNA-seq profiling of primary treatment-naïve and GCP-treated ICC samples. **(A, B)** Schematic diagram of study design and experimental flowchart. **(C)** tSNE plots showing cell origins from identified cell types (left) and treatment groups (right). **(D)** Box plots showing the inferred CNV scores of each cell type. **(E)** Multiplex immunofluorescence staining with anti-CD19, CD4, and CD8 antibodies in two resected ICC samples after GCP therapy. ICC, intrahepatic cholangiocarcinoma; GCP, gemcitabine-cisplatin chemotherapy plus anti-PD-L1; tSNE, t-distributed stochastic neighbor embedding; CNV, copy number variation.

To explore the cellular changes of ICC following GCP therapy, we collected samples from three ICC patients receiving GCP therapy for scRNA-seq analysis (**Figure 1A**). We further enrolled three cohorts to validate the major findings in the discovery cohort (**Figure 1B**). A tissue microarray (TMA) comprising 89 primary ICC specimens was constructed, and bulk RNA-seq data of 244 patients from the FU-iCCA cohort were analyzed. Moreover, we collected resected tumors from 32 additional patients who received surgical resection after GCP therapy (GCP cohort). Concordant with previous scRNA-seq findings in ICC (11, 30), 12 major cell types were identified based on highly expressed marker genes, including B cells, cholangiocytes, dendritic cells, endothelial cells, fibroblasts, hepatocytes, malignant cell, monocytes and macrophages, natural killer (NK) cells, plasmacytoid DCs (pDCs), plasma cells, and T cells (**Figure 1C**, **Supplementary Figure S1A**). We performed copy number variation (CNV) analysis to distinguish malignant cells from non-malignant cells (**Figure 1D**, **Supplementary Figure S1B**). H&E staining showed massive necrosis and fibrosis in the residual ICC area after GCP therapy (**Supplementary Figure S1C**). We also identified tertiary lymphoid structures (TLS) in the combination-treated, surgically resected tumors by multicolor IF staining (**Figure 1E**).

### 3.2 CCL18^+^ CD68^+^ macrophages are enriched in tumors and correlate with poor prognosis in ICC

**Figure 2:**
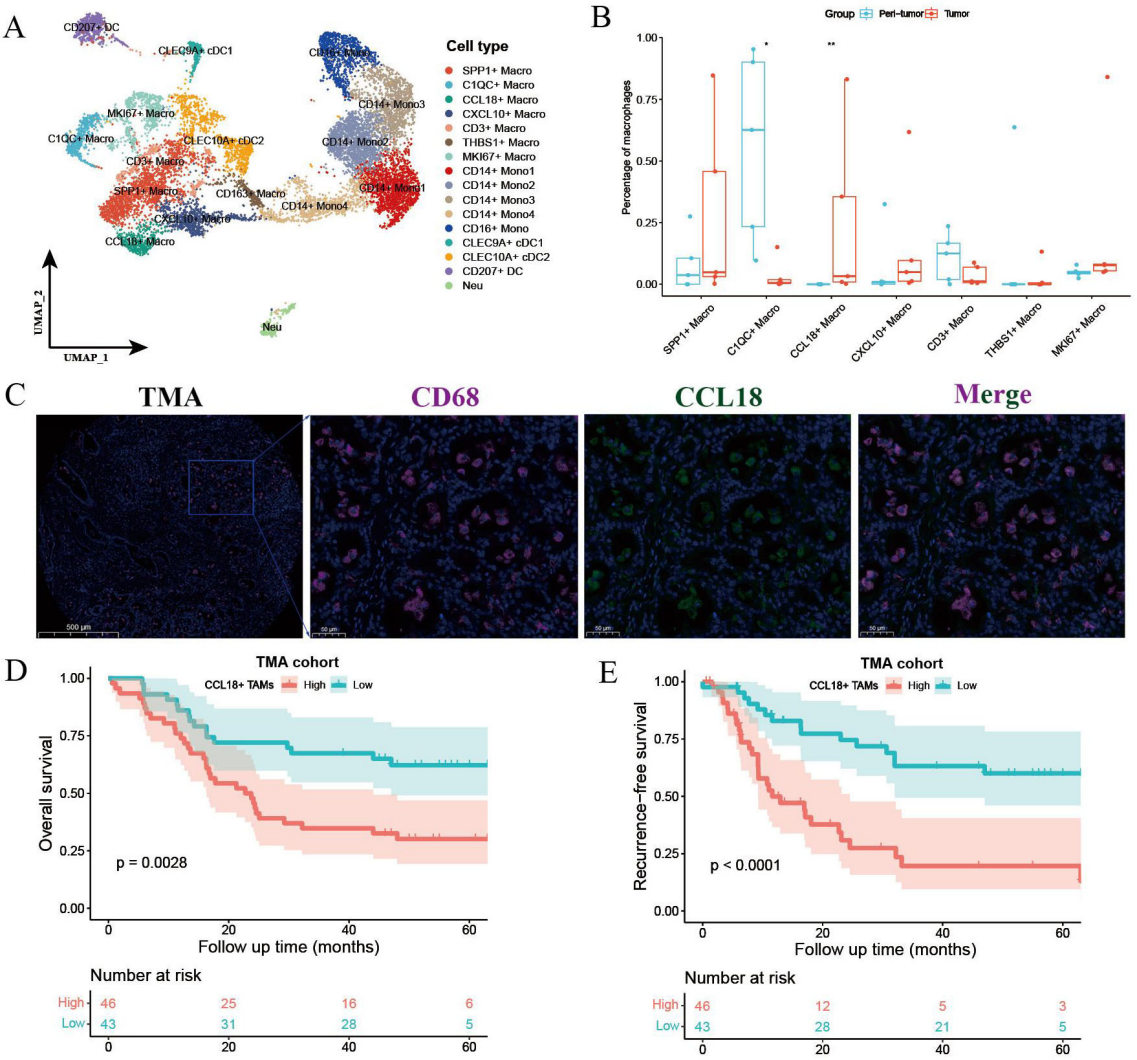
Myeloid cell components in primary treatment-naïve and GCP-treated ICC. **(A)** UMAP plot showing the subtypes of myeloid lineage cells. **(B)** Boxplot showing the fractions of macrophage subtypes in peri-tumor and tumors. Significance was determined by paired Wilcoxon test. **(C)** Representative immunofluorescence images of CCL18^+^ CD68^+^ macrophages in treatment-naïve ICC samples from TMA cohort. Kaplan–Meier survival curves for OS **(D)** and RFS **(E)** of 89 ICC patients grouped by cell counts of CCL18^+^ CD68^+^ macrophages. P values were determined via log-rank test. OS, overall survival; RFS, recurrence-free survival. *p < 0.05, **P < 0.01.

Unsupervised clustering of myeloid lineage cells identified 16 clusters, including 7 clusters for macrophages, 5 for monocytes (**Figure 2A**). SPP1^+^ macrophages highly expressed SPP1, NUPR1, and APOC1. CCL18^+^ macrophages exhibited high expression of APOE, CCL18, APOC1, and CSTB (**Supplementary Figure S2A**). Notably, CCL18^+^ macrophages were preferentially enriched in tumors compared with paired peri-tumor tissues (**Figure 2B**). SPP1^+^ macrophages also exhibited high intra-tumoral infiltration and predicted poor prognosis in ICC (17). In contrast, C1QC^+^ macrophages were more abundant in adjacent non-tumor tissues. To investigate the clinical significance of CCL18^+^ macrophages, we conducted survival analysis using bulk RNA-seq data of 244 ICC patients from the FU-iCCA cohort. Patients with a higher expression of CCL18 alone or the gene signatures of CCL18^+^ macrophages normalized by CD68 were associated with worse overall survival (**Supplementary Figures S2B, C**). The existence of CCL18^+^ CD68^+^ macrophages and such unfavorable prognostic patterns were further validated in the TMA cohort by IF staining (**Figures 2C-E**).

### 3.3 Tumor-infiltrating macrophages underwent antitumor remodeling following GCP treatment

**Figure 3:**
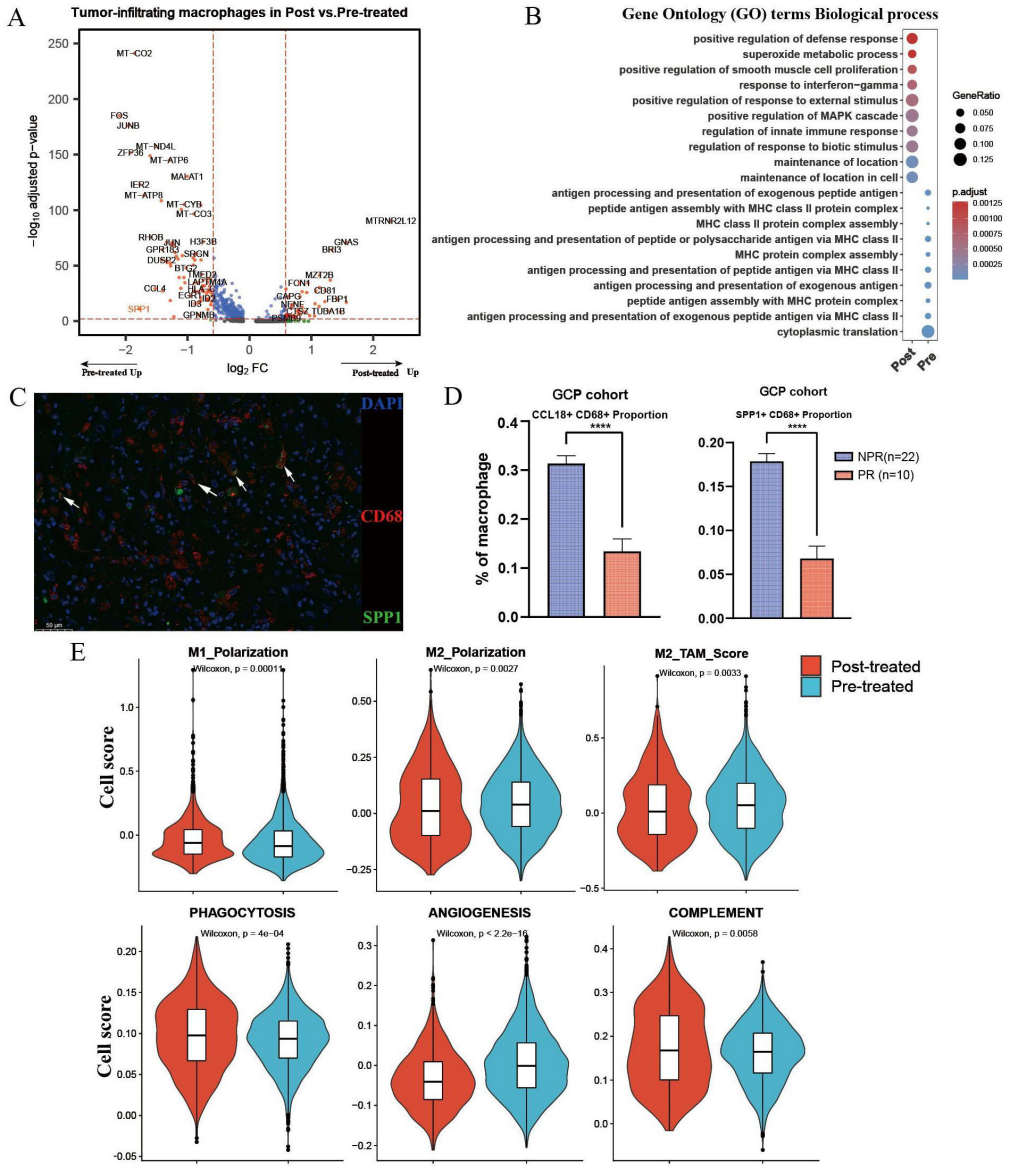
Differential analysis of macrophages in primary untreated and GCP-treated ICC samples. **(A)** Volcano plot showing differentially expressed genes of tumor-infiltrating macrophages between pretreated and posttreated ICC samples. **(B)** Bubble chart showing the distinct biological process of macrophages between pretreated and posttreated ICC samples revealed by Gene Ontology (GO) analysis. **(C)** Multiplex immunofluorescence of SPP1^+^ CD68^+^ macrophages in GCP-treated tumors from GCP cohort. **(D)** Boxplots showing cellular proportions of CCL18^+^ CD68^+^ (left) and SPP1^+^ CD68^+^ (right) macrophages in GCP-treated tumors evaluated by multiplex immunofluorescence. P values were determined by Wilcoxon test. **(E)** Phenotypic scores (M1 polarization, M2 polarization, M2-TAM score, phagocytosis, angiogenesis, and complement signatures) of macrophages between untreated and GCP-treated ICC samples. Significance was determined by unpaired two-tailed Wilcoxon rank-sum tests. ****p < 0.0001.

We further performed differentially expressed gene (DEG) analysis of intra-tumoral macrophages before and after GCP treatment (**Figure 3A**). Notably, SPP1, a well-documented oncogene in liver cancer, was significantly downregulated in macrophages after GCP therapy, in contrast to the upregulation following ICB monotherapy reported previously (17). Gene Ontology (GO) analysis (**Figure 3B**) showed that genes enriched in treatment-naïve macrophages were involved in antigen processing and presentation, whereas genes upregulated in GCP-treated macrophages were associated with immune response-related pathways (e.g., response to interferon-gamma, regulation of innate immune response). Moreover, IF staining of the GCP-treated, surgically resected tumors from the GCP cohort revealed that pathological responders harbored decreased infiltration levels of CCL18^+^ and SPP1^+^macrophages compared with non-responders (**Figures 3C, D**). To illustrate the phenotypic changes of macrophages in response to GCP, we calculated the classical phenotypic scores of macrophages such as M1 polarization, M2 polarization (28), phagocytosis, angiogenesis, and complement. Following GCP treatment, macrophages displayed significantly decreased scores of M2-like macrophage gene signatures, M2 polarization, and angiogenesis. Meanwhile, increased phenotypic scores of pro-M1 polarization, phagocytosis, and complement activity were observed in response to GCP (**Figure 3E**). These findings collectively suggested that GCP treatment could skew tumor-infiltrating macrophages from pro-tumor M2 toward antitumor M1-like phenotypes.

### 3.4 Tumor-infiltrating CD8^+^ T cells showed enhanced co-stimulatory signals in response to GCP therapy

**Figure 4:**
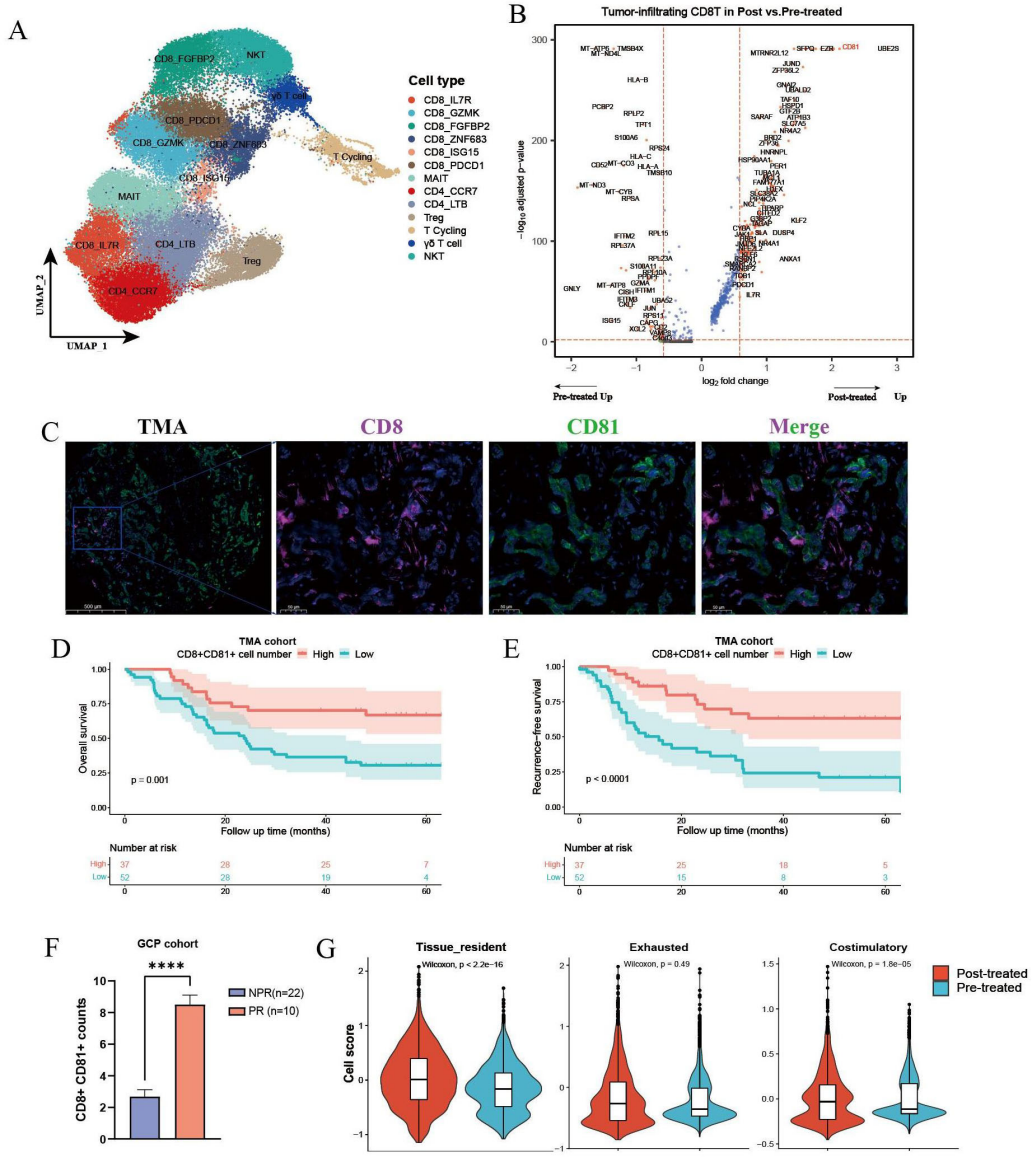
Differential analysis of CD8^+^ T cells between untreated and GCP-treated ICC samples. **(A)** UMAP plots showing all T cells labeled in different colors according to cell type annotation. **(B)** Volcano plot showing the differentially expressed genes of intra-tumoral CD8^+^ T cells between pretreated and posttreated ICC samples. **(C)** Immunofluorescence images showing the infiltration of CD8^+^CD81^+^ T cells in treatment-naïve ICC samples from the TMA cohort (n=89). Kaplan–Meier survival curves for OS **(D)** and RFS **(E)** of 89 ICC patients grouped by infiltration levels of CD8^+^ CD81^+^ T cells. P values were determined via log-rank test. **(F)** Boxplot showing infiltration levels of CD8^+^ CD81^+^ T cells in GCP-treated tumors revealed by multiplex immunofluorescence in GCP cohort. **(G)** Violin plots showing the expression scores of tissue resident, exhausted, and co-stimulatory gene signatures in tumor-infiltrating CD8^+^ T cells between pretreated and posttreated samples. The P values were calculated by Wilcox test. OS, overall survival; RFS, recurrence-free survival. ****p < 0.0001.

The efficacy of cancer immunotherapies depends in part on the revival of tumor-infiltrating T cells. Thus, we performed clustering analysis of all T cells and identified 13 subsets, including seven CD8^+^ subtypes and three CD4^+^ clusters (CD4_CCR7, CD4_LTB, and Treg) (**Figure 4A**, **Supplementary Figure S3A**). Differential analysis indicated that the top upregulated genes in CD8^+^ T cells from post treated tumors included UBE2S, CD81, DUSP5, TGFB1, and TNFSF9 (**Figure 4B**). GO analysis showed that the upregulated genes in post-treated CD8^+^ T cells were mainly enriched in pathways like T-cell activation and leukocyte cell–cell adhesion, whereas the biological processes of untreated CD8^+^ T cells were mainly involved in cytoplasmic translation, response to virus, and cytokine-mediated signaling pathway (**Supplementary Figure S3B**). CD81 mediates a complementary pathway for co-stimulation of T-cell activation besides CD28 (31). By performing IF staining in the TMA cohort, we found that the enrichment of CD81^+^ CD8^+^ T cells was associated with better survival outcomes in ICC patients (**Figures 4C-E**). DEG analysis showed that GCP-treated intra-tumoral CD4^+^ T cells also upregulated CD81, suggesting enhanced stimulatory signals for T cells (**Supplementary Figure S3C**). Moreover, CD81^+^ CD8^+^ T cells displayed higher infiltration levels in tumors achieving pathological response compared with those with non-pathological response (**Figure 4F**). To delineate the functional phenotypes of CD8^+^ T cells, we computed the expression scores of tissue resident, co-stimulatory, and exhausted gene signatures. GCP-treated CD8^+^ T cells harbored increased scores of tissue-resident and co-stimulatory molecules, compared with untreated CD8^+^ T cells, whereas exhausted scores showed no significant difference (**Figure 4G**).

### 3.5 Differential gene expression analysis of malignant cells before and after GCP treatment

**Figure 5:**
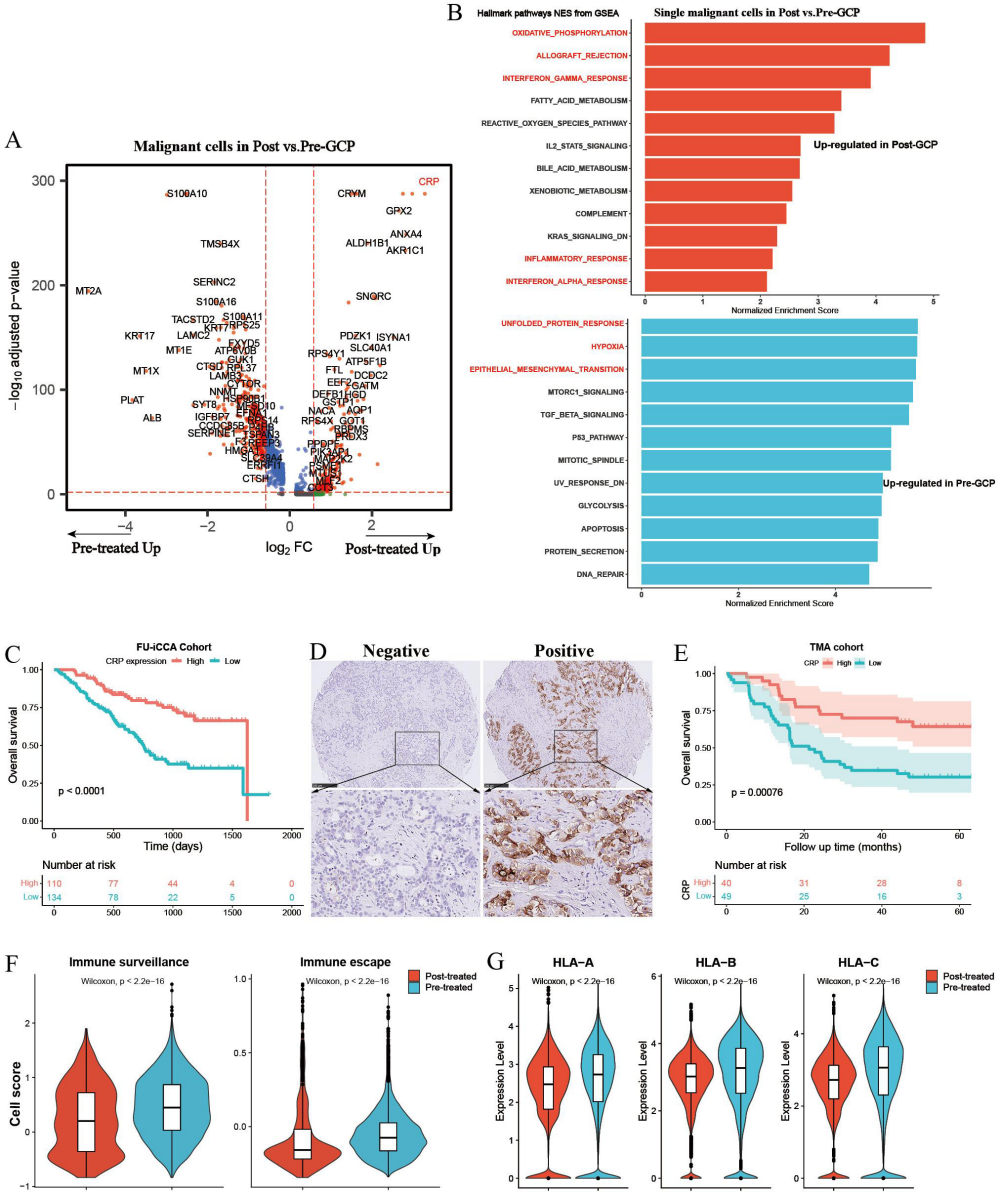
Differential gene expression analysis of single malignant cells in pretreated and posttreated ICC samples. **(A)** Volcano plot showing differentially expressed genes of malignant cells between pretreated and posttreated ICC. **(B)** Bar charts showing the upregulated Hallmark gene set pathways in GCP-treated and untreated malignant cells, respectively. **(C)** Kaplan–Meier survival curves of OS in the FU-iCCA cohort grouped by CRP mRNA expression level. **(D)** Representative immunohistochemistry images of CRP staining in ICC samples from the TMA cohort. **(E)** Kaplan–Meier survival curves for OS of 89 ICC patients after curative resection according to CRP expression levels in the TMA cohort. Violin plots showing the immune escape and immune surveillance scores **(F)**, as well as the expression levels of MHC-I genes (HLA/B/C) in malignant cells **(G)** from GCP-treated and pretreated samples. OS, overall survival; CRP, C-reactive protein; TMA, tissue microarray; MHC, major histocompatibility complex.

We further performed differential analysis between GCP-treated and untreated malignant cells (**Figure 5A**). Pathway enrichment analysis revealed an enrichment of genes in immune response pathways (e.g., allograft rejection, interferon gamma response, complement, inflammatory response) and metabolism-related pathways (e.g., oxidative phosphorylation, fatty acid metabolism, reactive oxygen species pathway, and bile acid metabolism) in GCP-treated malignant cells, whereas genes highly expressed in pretreated malignant cells mainly belonged to canonical oncogenic pathways (e.g., unfolded protein response, hypoxia, and epithelial mesenchymal transition) (**Figure 5B**, **Supplementary Figures S4A, B**). It is noteworthy that C-reactive protein (CRP) exhibited the highest upregulation with nearly an eightfold change in malignant cells following GCP treatment. CRP was reported as a diagnostic marker for ICC, and serum CRP flare could predict the response to anti-PD1 treatment in non-small cell lung cancer (32, 33). In the FU-iCCA cohort, patients with high CRP expression had significantly better prognosis (**Figure 5C**). We further validated that high CRP protein levels indicated better overall survival in the TMA cohort by IHC staining (**Figures 5D, E**). GCP-treated malignant cells also presented lower immune surveillance and escape signatures and decreased expression levels of HLA/B/C (**Figures 5F, G**).

### 3.6 Comparison of cellular interaction between GCP-treated and untreated ICC

**Figure 6:**
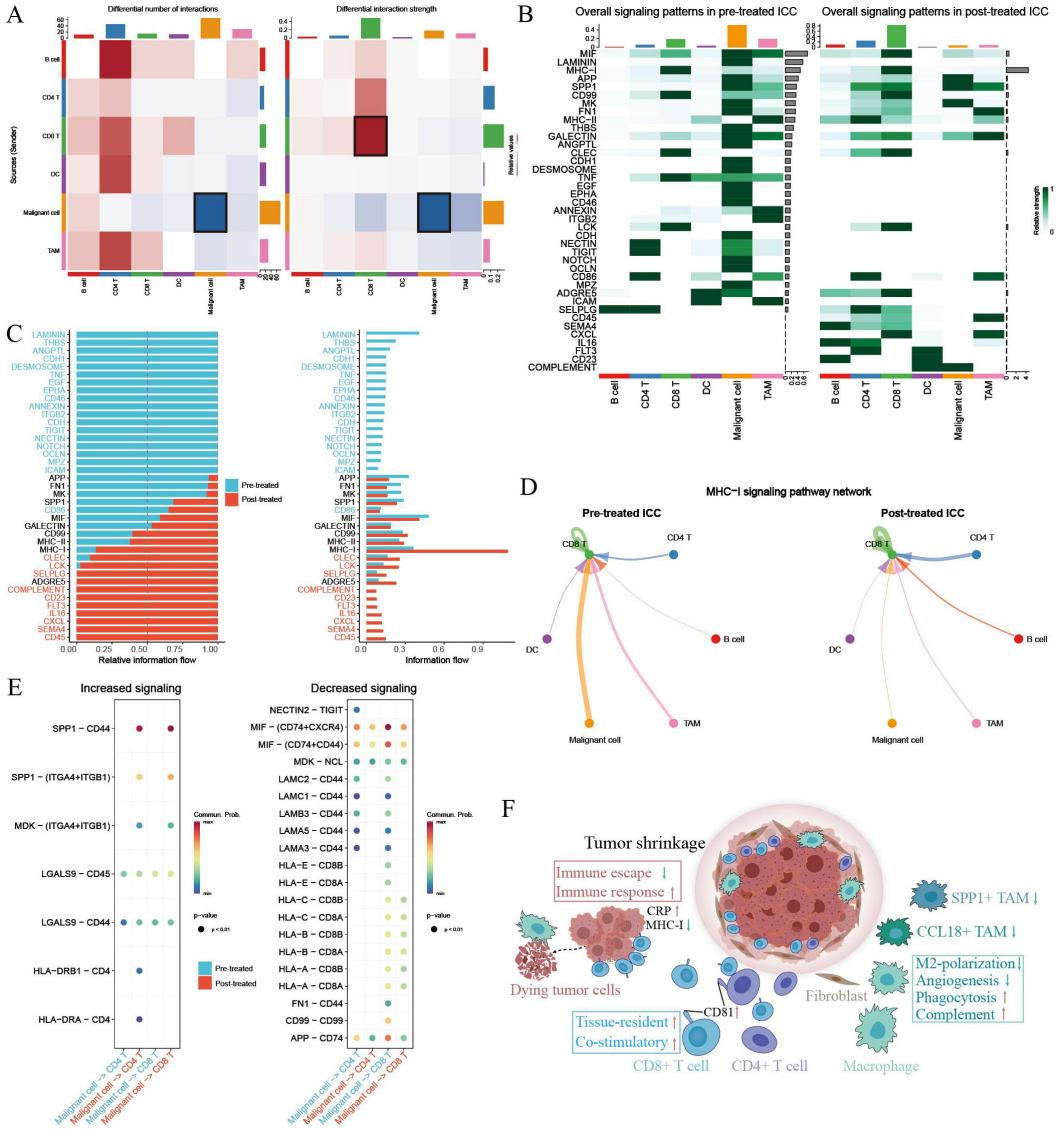
Comparison of cellular interactions between primary untreated and GCP-treated ICC ecosystems. **(A)** Heatmap showing the differential number (left) and strength (right) of cellular interactions between main cell types. Cell types are distinguished by colors. Red lines indicated increased number or strength of interactions, and blue lines suggested reduced number or strength. **(B)** Heatmap showing the specific signaling pathways of cell types in pretreated and posttreated ICC ecosystems. **(C)** Bar charts showing the differential overall signaling patterns in untreated and GCP-treated ICC ecosystem. **(D)** Circle plots showing the MHC-I signaling pathway networks in pretreated (left) and GCP-treated (right) ICC samples, respectively. **(E)** Dot plots showing increased (left) and decreased (right) signaling from malignant cells to CD4^+^ and CD8^+^ T cells, respectively. **(F)** Schematic diagram depicting intra-tumoral changes in response to GCP therapy.

To illustrate the cellular communication patterns following GCP therapy, we then compared the differential cell–cell interactions mediated by ligand–receptors between malignant cells and immune cells. Overall cell type interaction analysis exhibited a distinctive interplay in GCP-treated ICC ecosystem (**Figure 6A**). For example, increased interaction strength of CD8^+^ T cells and decreased interaction of malignant cells were observed following GCP treatment. Comparison of overall signaling patterns in GCP-treated and untreated ICC ecosystems revealed shared signaling pathways like APP, SPP1, and MIF. There were also several exclusive pathways in GCP-treated ICC, including complement, CD23, FLT3, IL16, CXCL, SEMA4, and CD45 (**Figures 6B, C**). Intriguingly, while the overall MHC-I signaling was increased in treated ICC, the MHC-I signaling between malignant cells and CD8^+^ T cells was decreased (**Figures 6C-E**), consistent with the reduced expression levels of MHC-I molecules in GCP-treated malignant cells (**Figure 5G**).

## 4 Discussion

Despite the rapidly evolving systemic therapeutic agents, the clinical outcomes of ICC patients remain unsatisfactory. We previously dissected the intra-tumoral changes in ICC following ICB therapy via single-cell analyses, highlighting the strategy of targeting SPP1^+^ macrophages and VEGF signaling (17). In this study, we delved deeper into mapping the cellular changes of ICC following GC chemotherapy combined with anti-PD-L1 therapy. Our findings reveal a reinvigorated antitumor immunity within the ICC ecosystem after GCP therapy. This revitalization was characterized by reduced fractions of CCL18^+^ and SPP1^+^ macrophages alongside antitumor phenotypic remodeling of tumor-infiltrating macrophages and CD8^+^ T cells (**Figure 6F**). Simultaneously, malignant cells displayed diminished immune evasion properties, coupled with inflammatory responses evidenced by elevated CRP expression.

It was reported that CCL18^+^ macrophages and SPP1^+^ macrophages were abundant in liver metastatic colorectal tumors and effective neoadjuvant chemotherapy (NAC) could eliminate them (34). We corroborated in this study that high intra-tumoral infiltration of CCL18^+^ CD68^+^ macrophages correlated with poor prognosis in ICC patients. In addition, reduced fractions of CCL18^+^ and SPP1^+^ macrophages were observed in those GCP-treated, surgically resected, and pathological responsive tumors. Differential gene expression analysis also revealed that SPP1 was downregulated in tumor-infiltrating macrophages after GCP treatment. Moreover, macrophages showed decreased expression of M2 polarization signature genes, concomitant with increased M1 polarization and phagocytosis activity. In comparison, we previously found that SPP1 expression was remarkably upregulated, and M2-like gene signatures were enriched in macrophages following ICB therapy (17). These findings may uncover the distinctive effects of ICB therapy alone or in combination with GC chemotherapy on intra-tumoral macrophages.

In our analyses, GCP-treated intra-tumoral CD8^+^ T cells harbored increased expression scores of both tissue-resident and costimulatory signatures. Notably, the expression of the CD81 molecule on both tumor-infiltrating CD8^+^ and CD4^+^ T cells was significantly enhanced following GCP combination therapy, emphasizing the potential role of CD81 in T-cell reactivation. CD81 functions independently of CD28 as a co-stimulator for both CD4^+^ and CD8^+^ T cells and preferentially activates naive T cells (31, 35). Moreover, CD81 co-stimulation of naive T cells prior to CAR transduction could lead to enhanced CAR expression in this T-cell subset (36). Collectively, our findings suggested that CD81 upregulation could mediate the enhanced stimulatory signals for T-cell activation against ICC tumors during GCP therapy.

Several limitations of this study should be acknowledged. First, due to the limited number of advanced ICC patients receiving surgical resection after GCP therapy, our study initially only included a relatively small patient sample size for scRNA-seq analysis. Although we subsequently recruited 32 additional patients to expand the GCP cohort and strengthen the validation of the scRNA-seq findings, the overall sample remains modest and may still limit the generalizability of the results. Future multicenter studies with larger cohorts are needed to further confirm these observations. Second, the precise mechanism by which GCP combination therapy induces antitumor phenotypic changes in immune cells within the ICC microenvironment remains unclear. While our data suggest a shift in immune cell profiles, the molecular pathways and cellular interactions underlying these changes have not been fully elucidated. Additional experiments, such as *in vivo* animal models, coculture systems, and detailed molecular analyses, will be essential to uncovering the causal relationships and biological processes involved. Lastly, the exploratory nature of part of our investigation necessitates cautious interpretation. Further functional validation and mechanistic studies are required to firmly establish the therapeutic potential of GCP and its possible synergies with existing treatments.

In summary, our preliminary findings reveal the beneficial effects of combined GC chemotherapy and anti-PD-L1 therapy on reshaping the ICC ecosystem and partially uncover the intricate intra-tumoral changes triggered by this effective combination regimen.

## Data Availability

The data reported in this paper have been deposited in the OMIX, China National Center for Bioinformation/Beijing Institute of Genomics, Chinese Academy of Sciences (https://ngdc.cncb.ac.cn/omix: accession no.OMIX013116).
