# Supplementary material for: Gemcitabine-cisplatin chemotherapy plus anti-PD-L1 therapy reinvigorates antitumor immune response by reprogramming the intrahepatic cholangiocarcinoma microenvironment
Source: Front Immunol. 2025 Dec 2;16:1666393. doi: 10.3389/fimmu.2025.1666393 (PMC12706460; doi:10.3389/fimmu.2025.1666393)
Supplement: Supplementary file 1 [file Table1.docx]

Supplementary Material

# **
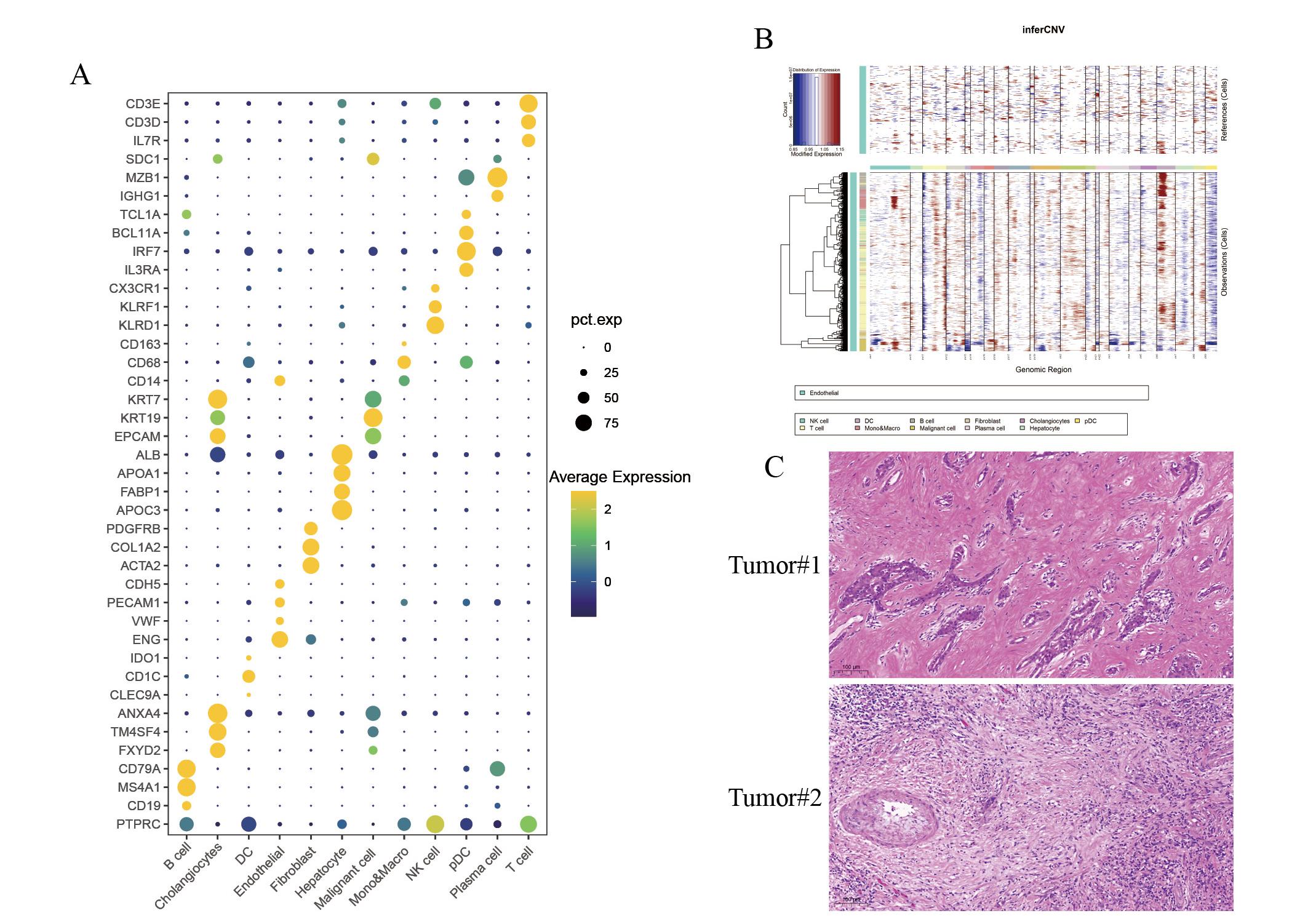
**Supplementary Figures

**Supplementary Figure 1. scRNA-seq profiling of primary untreated and GCP-treated ICC samples.**

1. Dot plot showing the expression of marker genes of the indicated cell types. **(B)** Heatmap showing the inferred copy number variation (CNV) analysis based on ScRNA-seq data, with endothelial cells set as normal reference. **(C)** H&E staining of resected ICC specimens showed the residual tumor area (×200).


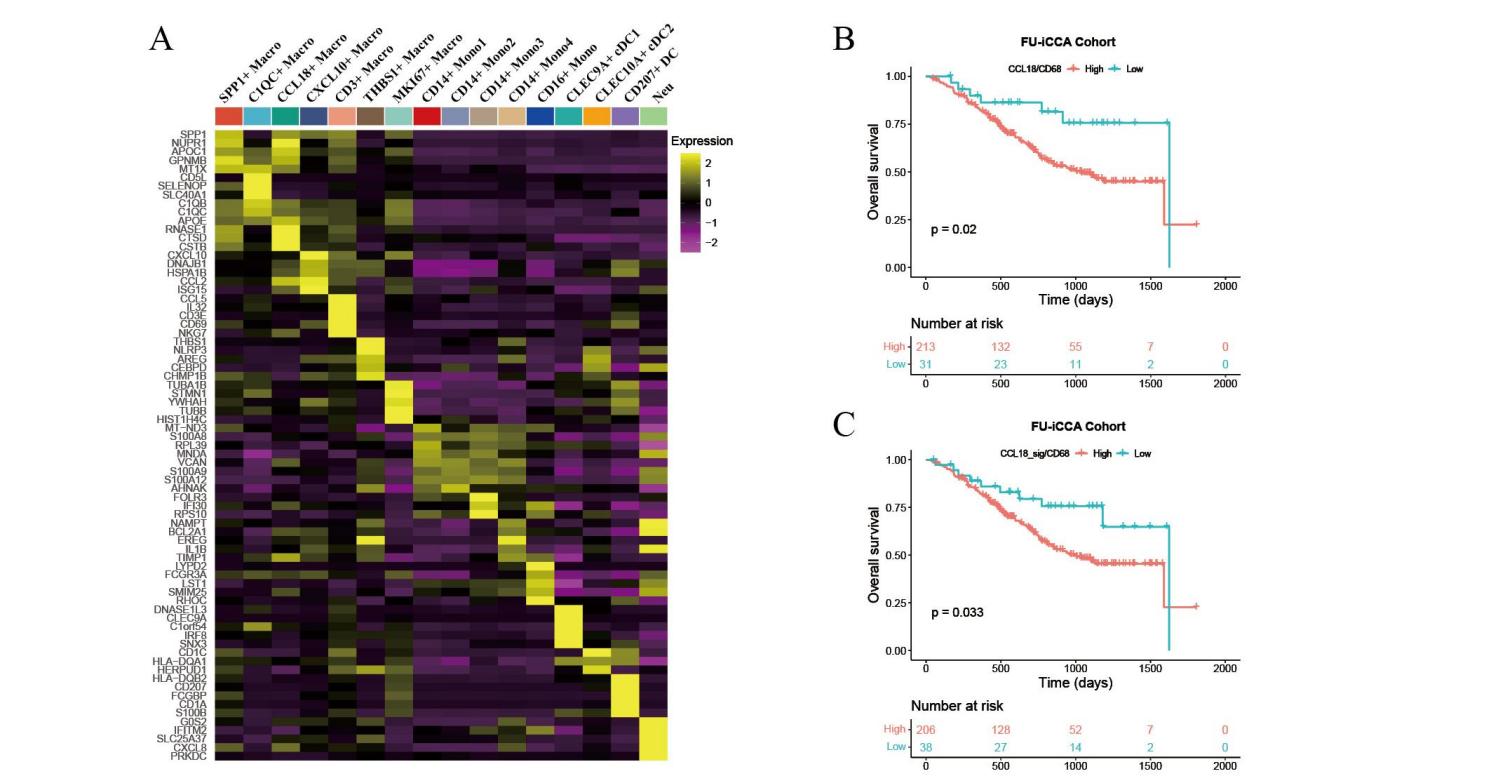


**Supplementary Figure 2. Myeloid cell components in primary treatment-naïve and GCP-treated ICC.**

**(A)** Heatmap showing the expression of top 5 highly expressed genes in each subset of myeloid cells. Kaplan-Meier survival curves showing that CCL18 **(B)** and CCL18^+^ macrophage **(C)** signatures were associated with worse OS in FU-iCCA cohort. OS, overall survival.

**Supplementary Figure 3.
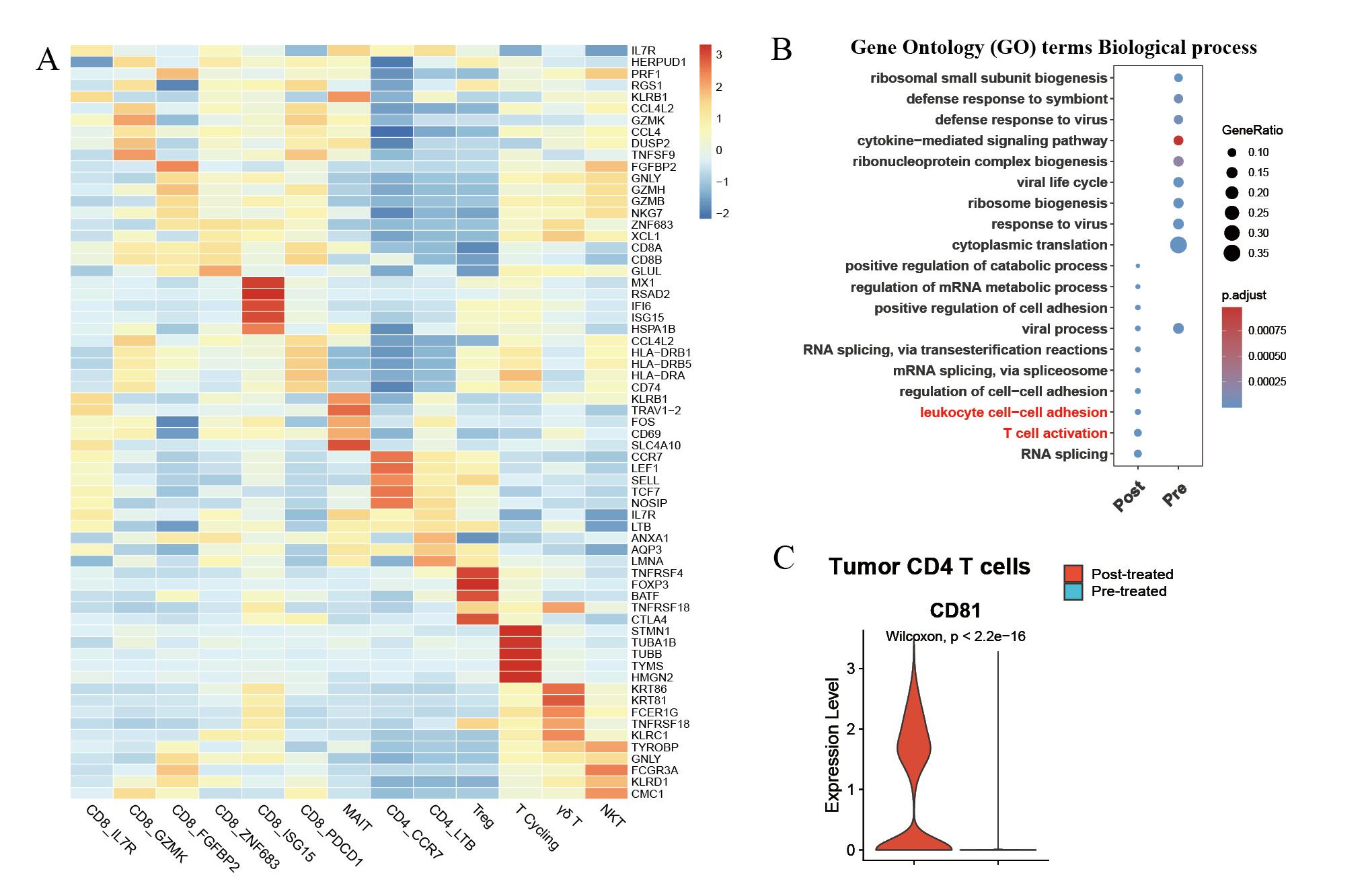
 Differential analysis of CD8^+^ and CD4^+^ T cells between primary untreated and GCP-treated ICC samples.**

**(A)**  Heatmap showing the expression of top 5 marker genes in indicated T cell subtypes. **(B)** Bubble chart showing the distinct biological process of tumor-infiltrating CD8^+^ T cells between pre-treated and post-treated samples by Gene Ontology (GO) analysis. **(C)** Violin plot showing the elevated expression of CD81 in CD4^+^ T cells from post-treated ICC samples compared with those from pre-treated samples.


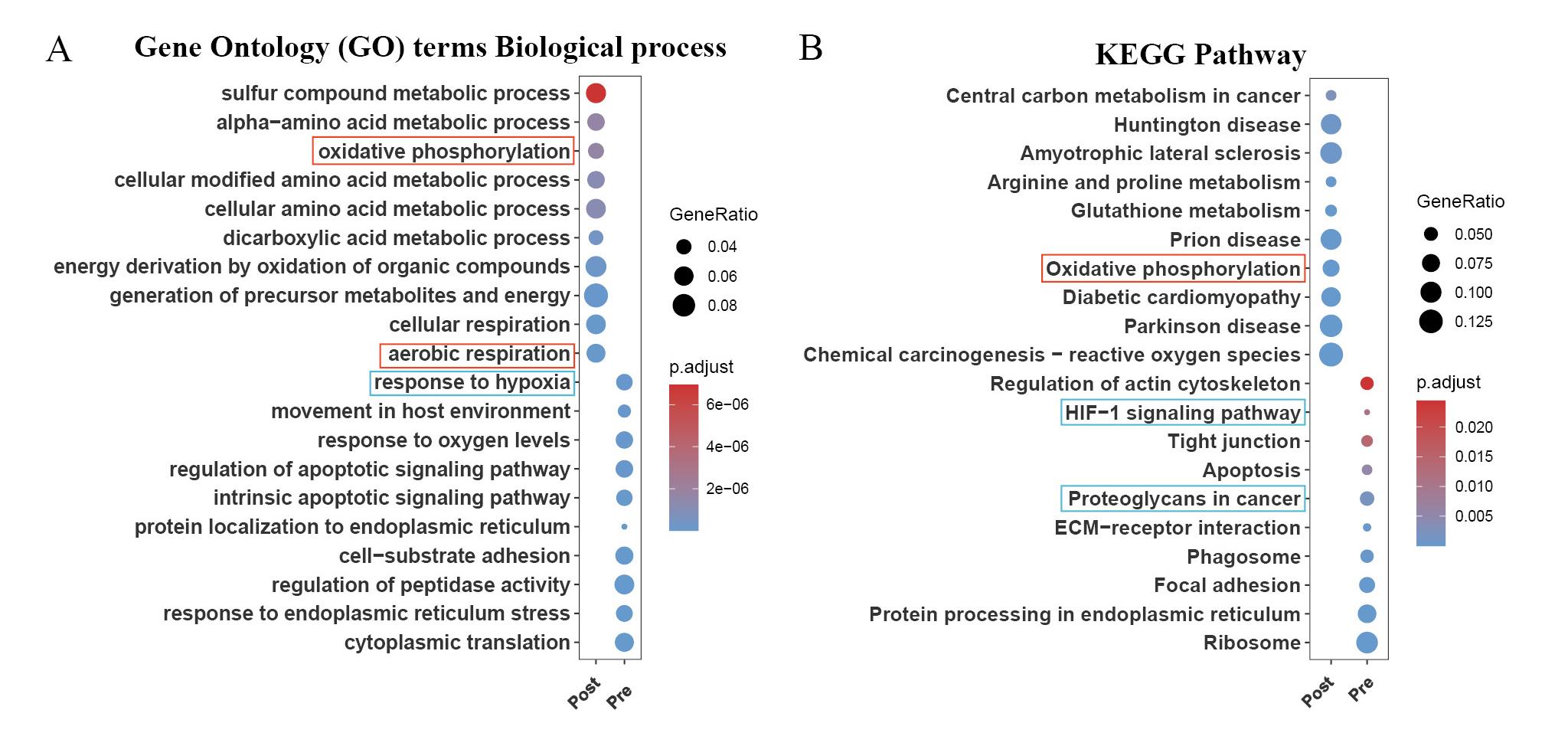
**Supplementary Figure 4. Differential gene expression analysis of single malignant cells before and after GCP therapy.**

**(A)** Bubble chart showing the enriched biological process in GCP-treated and untreated malignant cells revealed by Gene Ontology (GO) analysis. **(B)** Bubble chart showing the enrichment of KEGG pathways in post-treated and pre-treated malignant cells.
